# The Effect of Compliance With Preoperative Posturing Advice and Head Movements on the Progression of Macula-On Retinal Detachment

**DOI:** 10.1167/tvst.8.2.4

**Published:** 2019-03-26

**Authors:** Jan Hendrik de Jong, Koen de Koning, Tom den Ouden, Johan Casper van Meurs, Koenraad Arndt Vermeer

**Affiliations:** 1 The Rotterdam Eye Hospital, Rotterdam, The Netherlands; 2 Rotterdam Ophthalmic Institute, Rotterdam, The Netherlands; 3 Erasmus University Rotterdam, Department of Ophthalmology, Rotterdam, The Netherlands

**Keywords:** compliance, preoperative posturing, retinal detachment, progression, head motility

## Abstract

**Purpose:**

The aim of this study was to explore the relationship between compliance with preoperative posturing advice and progression of macula-on retinal detachment (RD) and to evaluate whether head positioning or head motility contributes most to RD progression.

**Methods:**

Sixteen patients with macula-on RD were enrolled, admitted to the ward, and instructed to posture preoperatively. The primary outcome parameter was compliance, which was defined as the average head orientation deviation from advised positioning. Secondary outcome parameters included the average rotational and linear head acceleration. The head orientation and acceleration were measured with a head-mounted inertial measurement unit (IMU). Optical coherence tomography (OCT) imaging was performed at baseline and during natural interruptions of posturing for meals and toilet visits to measure RD progression toward the fovea.

**Results:**

The Spearman correlation coefficient with RD progression was 0.37 (*P* = 0.001, *r*_s_^2^ = 0.13) for compliance, 0.52 (*P* < 0.001, *r*_s_^2^ = 0.27) for rotational acceleration, and 0.49 (*P* < 0.001, *r*_s_^2^ = 0.24) for linear acceleration. The correlation coefficient between RD progression and rotational acceleration was statistically significantly higher than the correlation coefficient between RD progression and compliance (*P* = 0.034).

**Conclusion:**

The strength of the correlation between RD progression and compliance was moderate. However, the correlation between RD progression and rotational and linear acceleration was much stronger. Preoperative posturing is effective by reducing head movements rather than enforcing head positioning.

**Translational Relevance:**

Monitoring the efficacy of preoperative posturing in macula-on RD using OCT and IMU measurements shows that a new and combined application of these technologies leads to clinically relevant insights.

## Introduction

Retinal detachment (RD) is a progressive separation of the retina from the underlying retinal pigment epithelium that occurs in 12 to 18 per 100,000 people per year.^1^,^2^ Visual acuity may be severely affected if the RD extends to the macula.^3^–^5^ To prevent macular involvement, preoperative posturing is prescribed while patients are waiting for surgery. Patients with macula-on RD are prescribed bed rest to reduce head and eye movements and related fluid currents.^6^–^13^ Additionally, patients are positioned supine when RD is located in the superior quadrants of the retina and upright for RD in the inferior quadrants to address the effect of gravity. To improve the compliance with this posturing advice, in some clinics patients are hospitalized during the preoperative period. An alternative approach is to provide surgery on a 24-hour, 7-days-per-week basis. As both approaches are expensive policies, the understanding of the effectiveness of preoperative posturing warrants further study. Recently, we used optical coherence tomography (OCT) to demonstrate that preoperative posturing reduces the progression of macula-on RD by comparing posturing with interruptions for meals and other short breaks.^14^ However, the strength of the relationship between compliance to preoperative posturing and RD progression is as yet unknown.

Compliance with positioning advice has been quantified previously using gravity- and tilt-compensated sensors after macular hole surgery.^15^–^17^ In this study, we used such sensors to measure the head orientation as well as the head's rotational and linear motility in patients with macula-on RD. Because the density differences between the retina and subretinal fluid are rather small, we would expect gravity to play a limited role in the progress of RD.^18^ Therefore, we hypothesized that head movements and eye movements contribute more to progression of RD than does head positioning.

The primary aim of this study was to explore the relationship between compliance with the preoperative posturing advice and the progression of macula-on RD. The secondary objective was to evaluate whether head positioning or head motility contributes most to the progression of RD.

## Patients and Methods

### Study Design

This study was designed as an explorative cohort study with recordings of head orientation, head motility, and the distance between the RD border and fovea during preoperative posturing of patients with macula-on RD. The study was approved by the local internal review board of the Rotterdam Eye Hospital and the medical ethical committee of the Erasmus Medical Center, Rotterdam, The Netherlands (identifier, 2014-502; www.trialregister.nl identifier, NTR4884). The study evaluated a small cohort that was enrolled in addition to a larger prospective trial evaluating preoperative posturing.^14^ The recordings of head orientation and head motility were performed only in the patients enrolled in the small additional cohort, which is presented in this report. All patients were hospitalized and examined in the Rotterdam Eye Hospital, Rotterdam, The Netherlands, and all provided written informed consent. The study was conducted in accordance with the tenets of the Declaration of Helsinki.

### Study Procedures

The study procedures were described previously in more detail.^14^ In brief, patients diagnosed with macula-on RD were admitted to the ward for posturing while they were waiting for surgery the same day, the next day, or occasionally the day after. Posturing consisted of two parts: bed rest and positioning. Patients with RD located mainly in the superior quadrant were positioned supine; patients with RD in the temporal quadrant on the temporal side of the affected eye, patients with RD in the nasal quadrant on the nasal side, and patients with RD in the inferior quadrant were instructed to sit upright. Patients were allowed to interrupt their posturing for meals, toilet visits, refreshment in the morning, and surgeon's examinations. Such intervals offer an excellent opportunity to acquire prospective and comparative data in an ethically acceptable manner.

### Inclusion and Exclusion Criteria

Inclusion criteria were age ≥ 18 years; written informed consent; nearest point of the RD border more than 1250 μm away from the foveola (safety measure) and within the range of the OCT system (estimated range was up to 10–12 mm from the fovea); sufficiently clear media to obtain an OCT scan; sufficient quality of the OCT scan; and OCT performed within an hour after admission of the patient to the ward. No exclusion criteria were specified. The safety border of 1250 μm from the foveola was defined by the traditional size of the fovea centralis (with a radius of approximately 750 μm) and parafovea (ring of 500 μm around the fovea) combined.^19^

### RD Progression Measurements

Within 1 hour after arrival on the ward, a baseline volume OCT scan (Widefield Spectralis OCT; Heidelberg Engineering, Heidelberg, Germany) was performed, and eligibility was determined. The distance between RD border and fovea was measured according to our previously described method.^14^ The 95% limits of agreement of the intrarater variability of these distance measurements was ±58 μm.^14^ The distance measurements on subsequent OCT scans were then used to calculate the RD border displacement and the average RD border displacement velocity (change in distance per hour) during posturing and interruption intervals. The latter measure adjusts for differences in interval duration and thereby enables a more consistent comparison between OCT measurements and average head orientation and head movements per measured interval. The average progression velocity from baseline was determined at each time point as well.

### Head-Mounted Inertial Measurement Unit

Measuring eye saccades over longer periods of time is not possible without invasive measures. However, measuring head orientation and motion is possible in a noninvasive manner by using a head-mounted electronic sensor, the Shimmer3 inertial measurement unit (IMU) (Shimmer Sensing, Glasnevin, Ireland). This IMU is small, lightweight, commercially available, and CE-marked, which indicates conformity with several health and safety regulations within the European Economic Area. After eligibility of a patient was determined, the IMU was fixed on the forehead of the patient with hypoallergenic, waterproof, and strongly adhesive plasters.

We configured the IMU to use three individual sensors: a low-noise accelerometer, a gyroscope, and a magnetometer at a 512-Hz sampling rate. The IMU was calibrated according to the north-west-up coordination system, which means that the *X*-axis points toward the north, the *Y*-axis toward the west, and the *Z*-axis up, perpendicular to the earth's surface. To prevent gimbal lock, quaternions were used instead of Euler angles to describe the three-dimensional rotations. The quaternions were calculated using the Shimmer Matlab Instrument Driver software (Shimmer Sensing), which estimates orientation data using magnetic angular rate and gravity (MARG) filtering. MARG filtering is reported to achieve orientation accuracy levels with less than 0.8° static error and less than 1.7° dynamic error.^19^

### Outcome Parameters

The primary outcome parameter of the IMU was defined as the orientation deviation from the advised positioning. We considered three secondary outcome parameters: the orientation deviation from the (presumed) optimal positioning, the rotational acceleration, and the linear acceleration.

#### Orientation Deviation From Advised Positioning

**Figure 1:**
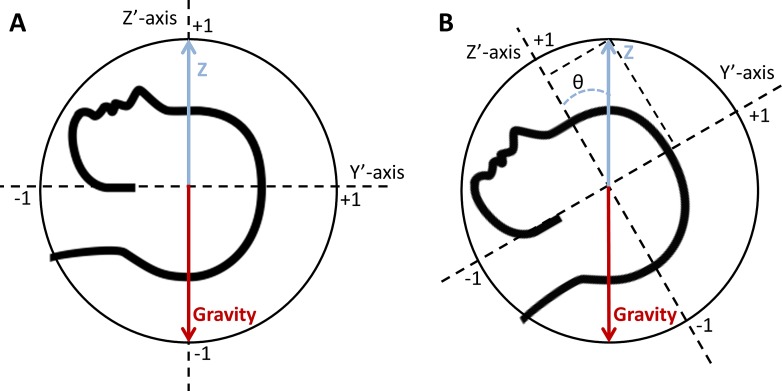
Schematic drawing of the position of the unit Z-vector (blue arrow) and gravity vector (red arrow) within the IMU coordinate system. When the patient is positioned supine with the IMU fixed on the forehead, the world Z-axis is aligned with the IMU Z′-axis (situation A). The coordinates of the unit Z-vector (blue arrow) on the IMU X′-, Y′- and Z′-axis will be 0, 0, and 1, respectively, in situation A. In situation B, a 30° rotation (θ) around the world X-axis has resulted in a 30° tilt of the IMU Y′- and Z′-axis and in a change of the coordinates of the unit Z-vector on the Y′-axis (this will be sin[θ]) and Z′-axis (cos [θ]). Rotation around the world Z-axis in either situation A or B will not change the coordinates of the unit Z-vector.

To obtain the deviation from the advised positioning, we used the relative (inverse) direction of gravity measured by the IMU at each time point. At the beginning of the first posturing interval, when the patient was positioned according to the advice, we determined the reference orientation of gravity (Fig. 1A). Subsequently, we used standard vector calculation to determine the smallest angle between the actual, measured gravity vector (Fig. 1B) and the reference gravity vector. Note that rotations around the gravity axis are not relevant and are ignored in this analysis. This transformed the orientation deviation, which could vary between 0° and 180°, into a compliance factor between 0 and 1, where 0 means perfect compliance and 1 means poor compliance.

#### Orientation Deviation From a Presumed Optimal Positioning

**Figure 2:**
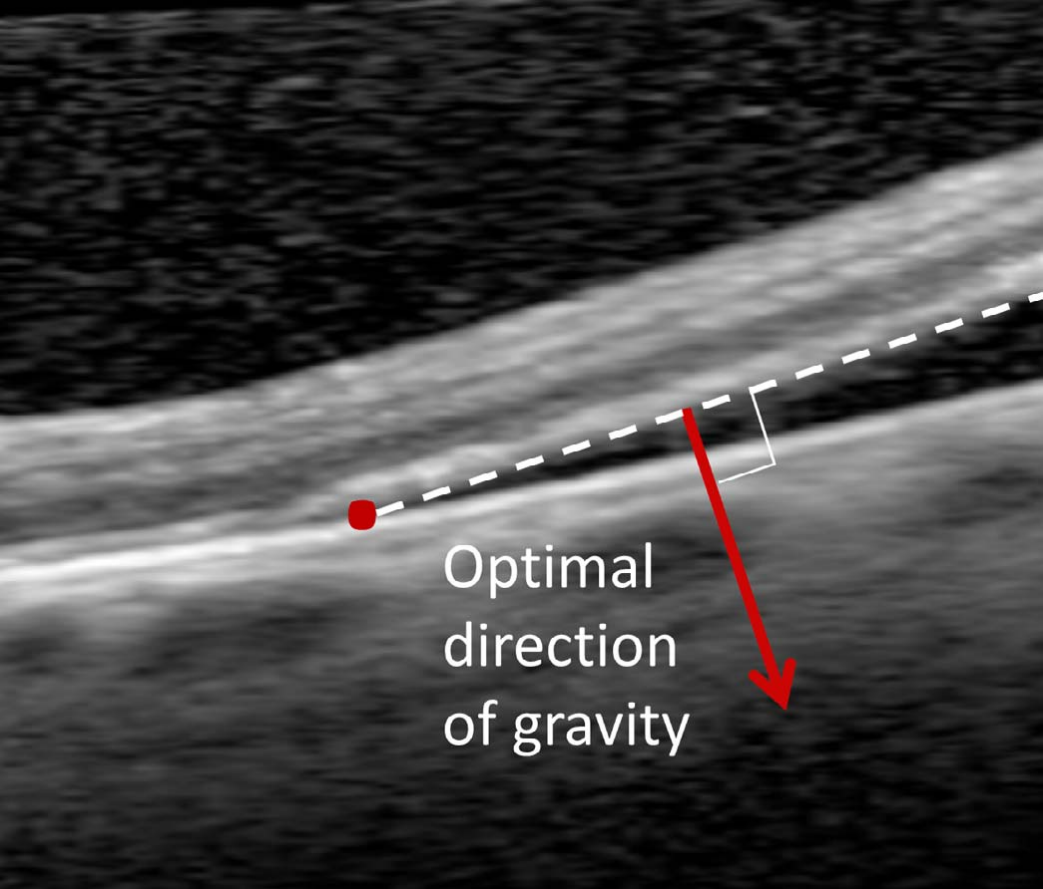
The optimal direction of gravity was defined as perpendicular to the detached retina closest to the fovea (red arrow).

Positioning is mostly prescribed in four categories: supine, temporal side, nasal side, and upright. This advice does not account for the distance between the fovea and the RD border or the precise location of the closest point on the RD border. For instance, positioning on the temporal side might be optimal for peripheral temporal RD, but a temporal RD that already has progressed close to the fovea might be better positioned supine to support reattachment of the retina closest to the fovea. Patients with inferior temporal RD might be better positioned with half-upright on the temporal side instead of a choice between temporal side or upright. We hypothesize that in optimal positioning the gravitation forces are directed perpendicular to detached retina that is closest to the fovea to facilitate reattachment of this part of the retina (see Fig. 2). Determining the presumed optimal position and using it as the reference position instead of the advised position might reveal whether patients would benefit from optimization of the posturing advice. A more detailed description of how this parameter was calculated can be found in Supplementary File S1.

**Figure 3:**
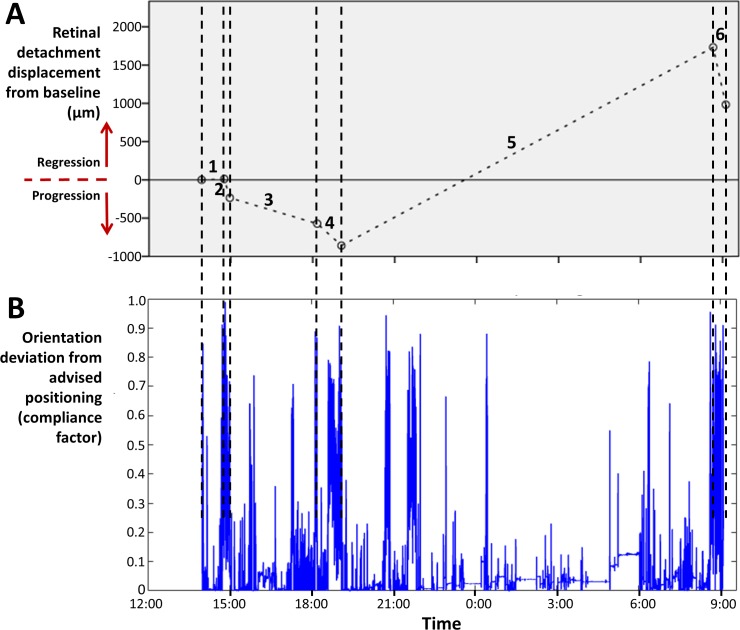
Example of the course of progression and the IMU parameters of a 56-year-old patient. The patient had an RD in the superior temporal quadrant of the right eye and supine posturing. During the day, most intervals showed progression, while during the night, regression of 2591 μm was seen (A). The orientation deviation was lower during posturing intervals than during interruptions, especially during the night (B, interval 5).

#### Rotational and Linear Acceleration

Accelerations around the X-, Y-, and Z-axes, both clockwise and counterclockwise, were all assumed to be equally relevant and included in the analysis. To obtain the rotational acceleration, the rotational velocity of two consecutive time points were subtracted from each other and divided by the time difference for all gyroscope axes separately. The total rotational acceleration was then defined by the root mean square of the rotational acceleration of the three axes.

To obtain the residual linear acceleration, we first corrected the measured linear acceleration for gravity. The total linear acceleration was then estimated by the root mean square of the residual linear accelerations obtained with the three accelerometer axes.

### IMU Parameter Outcome Per Interval

Because of the various durations of posturing and interruption intervals, the average of IMU parameter per interval was expected to provide the most consistent comparison to the average RD progression velocity per interval. The measured signal was corrected for the noise floor levels as seen during static test measurements and during the posturing intervals of the patient measurements.

We additionally wanted to determine whether head movements in general should be avoided by patients or whether sudden head movements with fast accelerations in particular should be avoided to prevent RD progression. Therefore, we determined the number of rotational and linear accelerations per interval per hour above a specific threshold and calculated the correlation with RD progression. We varied the threshold levels to evaluate whether higher thresholds would result in stronger correlation coefficients than the correlation coefficients between RD progression and the noise-corrected averages of IMU parameters. We varied the rotational acceleration thresholds between 250° and 10,000°/s^2^ at increments of 250°/s^2^ and the linear acceleration thresholds between 0.25 and 10 m/s^2^ at increments of 0.25 m/s^2^.

### Statistical Analysis

Due to the exploratory nature of this study, 16 patients with continuous measurements between admission to the ward and surgery were assumed to be sufficient to show general trends. We did not assume normally distributed data, and therefore nonparametric testing (Mann-Whitney *U* test) was used to compare RD progression and IMU parameters between posturing and interruption intervals. We expected a monotonic but possibly nonlinear relationship between RD progression and the IMU parameters. Therefore, Spearman's correlation coefficient was calculated to describe the relationship between RD progression and average IMU parameters. The correlation analysis was performed for all measured intervals as well as for the progression from baseline. For all IMU parameters, a positive correlation demonstrates an association with RD regression and a negative correlation an association with RD progression. Statistical significant differences between correlation coefficients were tested according to the methods of Meng et al.^20^ To determine whether the duration of follow-up (defined as the time between baseline OCT and last OCT measurement) influences the rate of RD progression from baseline, we also used the Spearman's correlation coefficient to describe the relationship.

## Results

### Patient Measurements and Example Patient

Sixteen consecutive patients were enrolled between December 7, 2016, and June 13, 2017. All patients were prescribed bed rest; three patients were positioned supine, seven on the nasal side, two on the temporal side, and four sitting upright. A total of 94 OCT scans was performed to record the RD displacement toward the fovea during 41 posturing intervals and 37 interruptions. The median duration of follow-up with OCT and the IMU was 18.1 hours (range, 2.1–35.7 hours). All patients provided a written informed consent. Patients' characteristics are summarized in Table 1.

**Table 1:** Patient and RD Characteristics^a^

Number of patients included in the study	16
Age, y	
Median (range)	56 (18–73)
Male:female, *N*	12:4
Phakic:pseudophakic, *N*	9:7
Snellen visual acuity	
Median (range)	20/25 (20/33–20/17)
*N* moderate myopia (≤6.0 D and ≥3.0 D)	3
*N* high myopia (≥6.0 D)	5
Duration of visual field loss, days	
Median (range)	5 (0.25–40)
No complaints of visual field loss, *N*	1
Primary:recurrent RD	14:2
History of vitrectomy	1
History of scleral buckling	1
Posterior vitreous detachment, yes/no	15
Extent of RD, degree	
Median (IQR)	62 (57–121)
Range	47–151
Size of retinal tear, *N*	
Single small, ≤0.50 clock h	2
Multiple/large, >0.50 clock h	12
No breaks found	2
Posturing advice, *N*	
Supine	2
Temporal side	3
Nasal side	7
Sitting upright	4
Baseline RD-fovea distance on OCT, μm	
Median (IQR)	6,535 (3,304–8,306)
Range	1,813–12,190
Time between baseline OCT and surgery, h	
Median (IQR)	21.3 (18.4–23.0)
Range	4.6–36.6
Time between baseline OCT and last OCT, h	
Median (IQR)	18.1 (13.3–19.4)
Range	2.1–35.7
Change of RD-fovea distance from baseline to the last OCT, μm	
Median (IQR)	−19 (−56 to 562)
Range	−847 to 1,934

a In patients with pseudophakic lens status, the spherical equivalent refraction before cataract surgery was used. D, diopter; IQR, interquartile range.

Figure 1 gives an example of the RD progression and head orientation deviation from the advised positioning of a patient with a superior temporal RD. This figure demonstrates that a larger orientation deviation results in more RD progression in this patient. During the day, there was moderate progression in both posturing intervals and fast progression during the interruptions. However, 2591-μm regression was seen during the posturing interval that included the night rest, and the lowest average IMU parameters where found during this interval as well, which demonstrates the efficacy of immobilization.

### RD Progression and IMU Parameters

A summary of the RD progression measurements is provided in Table 2. The median RD border displacement during posturing intervals was 10 μm (interquartile range [IQR]: −84 to 177 μm) and during interruptions −52 μm (IQR: −220 to 1 μm), which was statistically significantly different (*P* = 0.003). The median RD border displacement velocity during posturing intervals was −1 μm/h (IQR: −9 to 34 μm/h) and during interruptions −202 μm/h (IQR: −491 to 0 μm/h), which was statistically significantly different as well (*P* < 0.001).

**Table 2:** Comparison of RD Progression and IMU Outcome Parameters Between Posturing Intervals and Interruptions

	RD Border Displacement, μm	Duration, h	RD Border Displacement Velocity, μm/h	Average Orientation Deviation From Advised Positioning, Compliance Factor
Posturing intervals, *N* = 41				
Median (IQR)	10 (−84 to 177)	3.5 (1.8–11.4)	1 (−24 to 64)	0.04 (0.02–0.05)
Range	−538 to 2590	0.7–15.1	−147 to 871	0.01–0.20
Interruptions, *N* = 37				
Median (IQR)	−52 (−220 to 1)	0.4 (0.2–0.5)	−202 (−491 to 0)	0.30 (0.10–0.38)
Range	−749 to 96	0.1–1.0	−1625 to 227	0.01–0.52
Difference between posturing intervals and interruptions				
*P* value	0.002	<0.001	<0.001	<0.001

**Table 2:** Extended

	Average Orientation Deviation From Optimal Positioning, Optimal Compliance Factor	Average Rotational Acceleration, deg/s^2^	Average Linear Acceleration, m/s^2^
Posturing intervals, *N* = 41			
Median (IQR)	0.20 (0.10–0.37)	66 (56–97)	0.06 (0.04–0.10)
Range	0.04–0.56	43–193	0.01–0.33
Interruptions, *N* = 37			
Median (IQR)	0.46 (0.31–0.65)	181 (145–213)	0.26 (0.17–0.42)
Range	0.09–0.84	79–427	0.05–0.73
Difference between posturing intervals and interruptions			
*P* value	<0.001	<0.001	<0.001

The average IMU parameters for all posturing and interruption intervals, as well as the intervals from baseline, are described in Table 3. The applied noise thresholds were 1.8° for orientation deviation, 200 deg/s^2^ for rotational acceleration, and 0.8 m/s^2^ for linear acceleration. The difference between posturing intervals and interruptions was statistically significant for all four IMU parameters (*P* < 0.001).

**Table 3:** Correlation Analysis of Four IMU Outcome Parameters Against RD Border Displacement Velocity

	Average Orientation Deviation From Advised Positioning, Compliance Factor	Average Orientation Deviation From Optimal Positioning, Optimal Compliance Factor
Posturing intervals and interruptions, *N* = 78		
Correlation with RD border displacement velocity		
Spearman's ρ (95% CI)	−0.37^a^ (–0.56 to −0.13)	−0.36^a^ (−0.53 to −0.14)
*P* value^b^	0.001	0.001
Difference between correlation coefficients, column 1 against 2, 3, and 4		
Spearman's ρ difference (95% CI)	NA	−0.01 (−0.24 to 0.22)
*P* value (single-sided)^b^		0.465
Change from baseline, *N* = 78		
Correlation with RD border displacement velocity		
Spearman's ρ (95% CI)	−0.06 (−0.30 to 0.19)	0.11 (−0.13 to 0.34)
*P* value^b^	0.58	0.35
Difference between correlation coefficients (column 1 against 2, 3, and 4)		
Spearman's ρ difference (95% CI)	NA	−0.17 (−0.45 to 0.14)
*P* value (single-sided)^b^		0.143

a This correlation is still statistically significant after Bonferroni correction for the 14 correlation analyses (*P* level 0.05/14 = 0.004),

b *P* value without Bonferroni correction.

**Table 3:** Extended

	Average Rotational Acceleration (deg/s^2^)	Average Linear Acceleration (m/s^2^)
Posturing intervals and interruptions, *N* = 78		
Correlation with RD border displacement velocity		
Spearman's ρ (95% CI)	−0.52^a^ (−0.68 to −0.30)	−0.49^a^ (−0.69 to −0.25)
*P* value^b^	<0.001	<0.001
Difference between correlation coefficients, column 1 against 2, 3, and 4		
Spearman's ρ difference (95% CI)	0.15 (−0.01 to 0.37)	0.12 (−0.03 to 0.32)
*P* value (single-sided)^b^	0.034	0.054
Change from baseline, *N* = 78		
Correlation with RD border displacement velocity		
Spearman's ρ (95% CI)	−0.36^a^ (−0.15 to −0.53)	−0.30 (−0.50 to −0.11)
*P* value^b^	0.001	0.007
Difference between correlation coefficients (column 1 against 2, 3, and 4)		
Spearman's ρ difference (95% CI)	0.29 (0.04 to 0.52)	0.24 (0.05 to 0.42)
*P* value (single-sided)^b^	0.012	0.008

### Correlation Analysis

**Figure 4:**
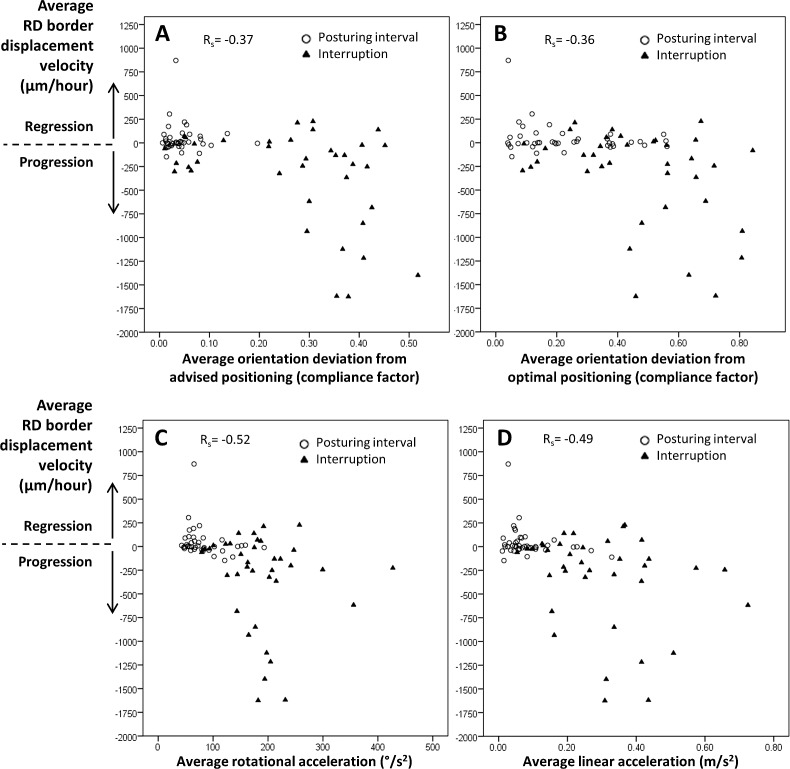
Scatter plots of the four IMU parameters with RD progression. RD progression was defined as the average RD border displacement velocity (μm/h) and calculated for posturing intervals (circles) and interruptions (triangles) separately. The scatter plots display the nonlinear and negative relationship between the four IMU parameters and RD progression. The strength of the Spearman correlation (R_s_) was moderate for the relationship between RD progression and average orientation deviation from advised positioning (A) and optimal positioning (B). The strength of the correlation between RD progression and rotational acceleration (C) and linear acceleration (D) was much stronger.

The correlations of RD border displacement velocity and the IMU parameters are provided in Table 3 as well. Figure 4 shows the scatter plots of RD progression and IMU parameters. The strongest Spearman's ρ correlation coefficients (*r*_s_) between RD progression and the IMU parameters were found for rotational acceleration (*r*_s_ = 0.52) and linear acceleration (*r*_s_ = 0.49). The *r*_s_^2^ can be interpreted as a proportion of explained variance if the IMU parameters and the RD progression are presented as ranked variables. The higher this proportion, the more variance is explained by a specific variable. The *r*_s_^2^ was 0.13 for orientation deviation from the advised positioning, 0.13 for the orientation deviation from optimal positioning, 0.27 for the rotational acceleration, and 0.24 for the linear acceleration. This means that rotational acceleration as well as linear acceleration seems to explain twice as much of the variance of RD progression than orientation deviation from advised or optimal positioning. The correlation coefficient between RD progression and rotational acceleration was statistically significantly higher than the correlation coefficient between RD progression and compliance (*P* = 0.034; see also Table 3).

The Spearman's correlation coefficient between orientation deviation from advised positioning with the other three IMU parameters was 0.52 (*r*_s_^2^ = 0.27) for orientation deviation from optimal positioning, 0.68 (*r*_s_^2^ = 0.46) for rotational acceleration, and 0.72 (*r*_s_^2^ = 0.49) for linear acceleration. This means that the secondary IMU parameters are codependent with the primary IMU parameter (orientation deviation from advised positioning), but they are not the same.

The average head orientation deviation was not significantly correlated with RD progression from baseline (see Table 3, row 2). However, RD progression from baseline was statistically significantly correlated with the average rotational acceleration (*r*_s_ = −0.36; *P* = 0.001).

The correlation between the duration of follow-up and the change of RD-fovea distance from baseline to the last OCT measurement was 0.08 (95% confidence interval [CI] −0.42 to 0.58; *P* = 0.76).

### Correlation With the Number of Accelerations per Interval

We studied the correlation between RD progression and the average number of accelerations above various thresholds per posturing and interruption interval. The strongest Spearman's correlation coefficient was 0.51 at a threshold level of 2000 deg/s^2^ for rotational acceleration and 0.51 at a threshold level of 1.25 m/s^2^ for linear acceleration (for full analysis, see Supplementary File S2). The increase of threshold levels did not result in substantially higher correlation coefficients than the correlation between RD progression and average IMU parameters per interval as presented in Table 3.

## Discussion

To our knowledge, the compliance with preoperative posturing advice in patients with macula-on RD and the correlation with RD progression has not been studied previously. We showed that the strength of the correlation between RD progression and head orientation deviation from advised and optimal positioning was moderate. However, the correlation of RD progression with rotational and linear acceleration was much stronger, both for the progression during posturing and interruption intervals and the progression from baseline. Therefore, we conclude that preoperative posturing is effective by reducing head movements rather than enforcing head positioning.

The clinical significance of the strong correlation between RD progression and head motility is that patients will benefit from moving their head as little as possible during the preoperative period. This can be accomplished by bed rest and by avoiding unnecessary activities involving head motion. Any required transportation may be done by bed or wheelchair (preferably with suspension) to minimize the amount of head and eye movements. Previous research showed that a reduction of eye movements (saccades) by double patching the eyes or suturing the eye muscles to the bulbus resulted in a reduction of subretinal fluid.^6^–^11^ Apparently, a reduction of head movements is also beneficial to prevent RD progression.

Several other factors may affect RD progression. Most importantly, we measured head movements, whereas saccades are traditionally expected to be able to overcome the forces of retinal adhesion.^21^,^22^ The rotational velocity and acceleration of saccades are typically faster than those of active head rotations.^23^–^27^ However, the radius of the head is greater than the radius of the eye, whereas the magnitude of saccades is smaller than that of head movements.^28^ Therefore, the tangential linear acceleration of the components of RD may be in the same range. During a head rotation, the movement of the eye approximates a translational movement. The direction of acceleration and deceleration forces of the fluids at opposite sides of the eye will be almost parallel during rotational head movements and precisely parallel during linear head movements. As a result, the effect on fluid currents within the eye may be limited. During a saccadic eye rotation, however, the direction of acceleration and deceleration forces will be opposite on the opposite sides of the eye, which is likely to create strong fluid currents of both liquefied vitreous and subretinal fluid. Nevertheless, the number and strength of saccades can partly be predicted by the number and strength of head movements as measured in the current study.^29^,^30^ Therefore, if saccades were to be measured independently from head movements, we expect that only a small additional part of the variance of RD progression could be explained.

There are at least four other factors that may play a role in RD progression. Firstly, the retinal adhesion strength differs between retinal locations and is especially higher at the macula. It is a common observation among surgeons that the peripheral retina detaches much more easily than does the posterior retina when creating a RD for macular rotation or retinal pigment epithelium–choroid graft, suggesting a difference in adhesion.^31^,^32^ We previously demonstrated that a small RD in the periphery has a higher progression risk, suggesting a difference in retinal adhesion as well.^14^ Secondly, the amount of subretinal fluid and the shape of the detachment differs between RDs. It is expected that the retina reattaches faster in a flat RD than in a bullous RD with the same area of detachment because the subretinal fluid volume is smaller and will be reabsorbed earlier.^12^,^33^–^36^ Thirdly, the size, number, and type of retinal breaks differ between RD patients, where a large horseshoe-shaped retinal tear is more likely to facilitate inflow of liquefied vitreous into the subretinal space than do small round holes.^12^,^13^,^37^ Finally, the contractile properties of the detached, incompletely detached, or not detached vitreous differs among patients, mostly due to the effects of aging of the vitreous.^38^,^39^ Progressive traction of contractile vitreous may detach the retina surrounding the retinal break, allowing more liquefied vitreous to enter the subretinal space.^12^,^13^ Because of all these factors, head and eye movements can be only partly accountable for the variance in RD progression.

Evaluation of the orientation deviation from optimal positioning did not reveal a stronger correlation with RD progression than did the orientation deviation from advised positioning. This suggests that the optimization of positioning would not significantly reduce RD progression. It also suggests that the role of gravity is limited, which is expected because the density difference between the retina and subretinal fluid is small.^18^

Evaluation of the number of accelerations per interval above various thresholds did not reveal a substantially higher correlation with RD progression than did the average of IMU parameters per interval. This might indicate that relatively slow head accelerations are also able to induce RD progression, or it might be that patients did not frequently perform sudden head movements during their hospitalization and the number of fast head accelerations was too low to reveal a stronger relationship. We cannot conclude that only strong or sudden head movements should be avoided. Evaluation of the relationship between the duration of follow-up and the change of RD-fovea distance from baseline did not reveal a statistically significant relationship. As pointed out above, RD progression can be explained by other factors only than the duration of follow-up.

Our method by which we measured head orientation might be used, in combination with OCT distance measurements, to evaluate the effect of delayed surgery for 1 day with preoperative posturing at home. This alternative policy might be cost saving for both clinics that aim to provide 7 days per week surgery service and clinics that hospitalize patients preoperatively. However, such a study should take into account the expected differences in characteristics and behavior between hospitalized patients and patients who are asked to stay quiet at home. IMU devices might also be used for other areas of ophthalmology where the effect of posturing regimes warrants validation, such as postoperative positioning after macular hole surgery,^15^–^17^ after RD surgery when intraocular gas is used, after pneumatic displacement of submacular hemorrhages, and after corneal transplantation when air bubbles are used to facilitate attachment of the graft.

Strengths of this study include the objective measurements of head orientation, head movements, and RD progression and the reasonable amount of 78 monitored intervals. Limitations include the small number of patients, the variation in RD localization and subsequent positioning advice, and the differences in follow-up duration. In addition, the patient might have touched the device causing false rotational and linear accelerations. Since this would result only in short acceleration peaks, we think that the influence on the averages and number of accelerations per interval is small.

In conclusion, preoperative posturing advice should emphasize a reduction of head movements, although positioning might be beneficial to prevent RD progression as well. This study may be an important step toward an evidence-based policy for optimal preoperative posturing in patients with macula-on RD.

## Supplementary Material

Supplement 1

Supplement 2
