# Supplementary material for: The Effect of Compliance With Preoperative Posturing Advice and Head Movements on the Progression of Macula-On Retinal Detachment
Source: Transl Vis Sci Technol. 2019 Mar 26;8(2):4. doi: 10.1167/tvst.8.2.4 (PMC6438104; doi:10.1167/tvst.8.2.4)
Supplement: Supplement 1 [file tvst-08-02-02_s01.pdf]

Supplemental Digital File 1

Description of the calculation of orientation deviation from optimal positioning

To determine the optimal unit Z-vector we used the OCT scan to measure the angle between the RPE and retina in the part of the detachment closest to the fovea (see Supplemental Figure 1A). We assumed that according to the standard orientation of the IMU on the forehead of the patient, the IMU Z' axis would be aligned with the anterior-posterior axis of the eye. We also assumed that the average eye gaze direction was straight ahead during any position of the head. We calculated the angle between the presumed optimal vector and the Z'-axis using formula (1), see also supplemental figure 1.

$$\beta = 2 \cdot \arcsin\left(\frac{0{,}5 \cdot L}{R}\right) + \alpha \qquad (1)$$

Where β is the angle between the optimal vector and the Z'-axis in degree, L is the shortest distance between the fovea and the RD border in mm, R is the radius of the eye, which was set to 12 mm for all patients and α is the angle between the retinal pigment epithelium and the retina in degree (see supplemental Figure 1A).

The coordinates of the unit Z-vector were then calculated for the situation that the patient would be positioned with the unit Z-vector pointing in the opposite direction of the direction of gravity using formula (2), see also supplemental figure 1.

$$\begin{bmatrix} Z_{X'} \\ Z_{Y'} \\ Z_{Z'} \end{bmatrix} = \begin{matrix} -\sin(\beta) \cdot \sin(\gamma) \\ -\sin(\beta) \cdot \cos(\gamma) \\ \cos(\beta) \end{matrix} \qquad (2)$$

Where $Z_{X'}$, $Z_{Y'}$, $Z_{Z'}$ are the X', Y' and Z'-coordinates of the unit Z-vector during a presumed optimal positioning (for an explanation of the unit Z-vector and IMU axis X', Y' and Z', see also Figure 1 in the manuscript), β the angle between the optimal vector and the Z'-axis in degree (derived from formula 1), γ the clockwise angle between 12 o-clock and the line between fovea and the closest point to the fovea on the RD border (in degree, see supplemental figure 1B).

The deviation from the presumed optimal positioning was then calculated for all time points in degrees and a cosine transformation (compliance factor = 0.5 - cos(angle)/2) was performed as well which resulted in a compliance factor between 0 and 1 (where 0 means perfect compliance and 1 means poor compliance).

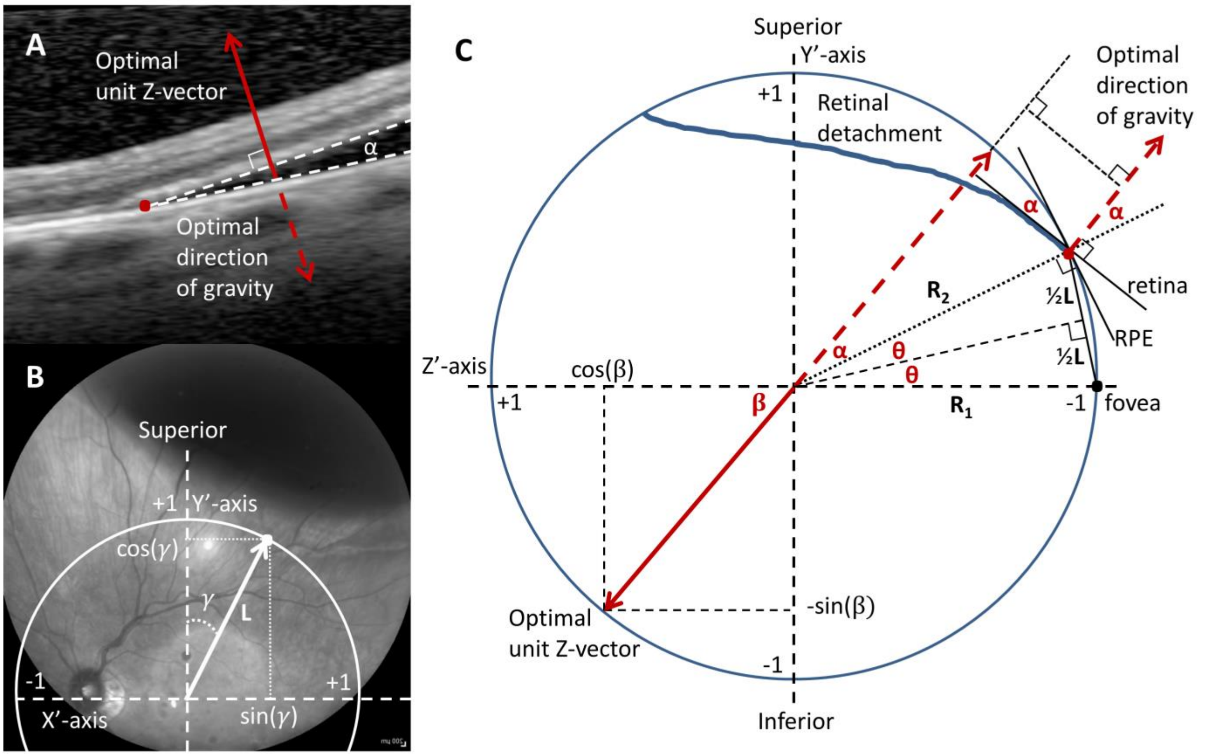

Supplemental figure 1. Definition of the coordinates of the presumed optimal unit Z-vector on the IMU X', Y' and Z'-axis. The optimal direction of gravity was defined as perpendicular to the first part of the detached retina in the direction of the retinal pigment epithelium (A, dashed red arrow) and the optimal unit Z-vector as the inverse vector (A, red arrow) .

To determine the coordinates of the presumed optimal unit Z-vector, we first defined the angle between the Z'-axis and the normal (C, R_2) of the closest point to the fovea on the RD border (C, red dot).The distance between the fovea (C, black dot) and the closest point to the fovea on the RD border (C, red dot) was measured with OCT and is called L (see B and C). This line L forms a isosceles triangle with the Z' axis (C, R_1) and the black dotted normal-line (C, R_2). If we bisect this triangle, we can calculate θ by the following formula: $\theta = \sin^{-1} (0.5 \times L / R)$, where R is the radius of the eye, which was defined as 12 mm for all patients.

Next step was to rotate the normal further towards the direction of gravity by angle α. We assumed that the tangents of the closest point on the RD border (C, red dot) closely resembles the curvature of the RPE in the first part of the detachment (A, lower dashed line and C, RPE-line).Since the RPE-line has an angle of 90 degrees with the normal (C,RPE-line and R_2), the normal has to rotate with angle α to find the presumed optimal direction of gravity. The angle β between the Z'-axis and the unit Z-vector was then calculated with the following formula: $\beta = 2 \times \theta + \alpha = 2 \times \sin^{-1} (0.5 \times L / R) + \alpha$. The Z' coordinate of the unit Z-vector was then defined as $Z_{Z'} = \cos(\beta)$. To find the X' and Y' coordinates, we measured the deviation γ of L from the superior or 12 o-clock line (see γ and L in B). Then the X' coordinate of the unit Z-vector could be defined as $Z_{X'} = -\sin(\beta) \times \sin(\gamma)$ and the Y' coordinate as $Z_{Y'} = -\sin(\beta) \times \cos(\gamma)$.
