# Supplementary material for: The Effect of Compliance With Preoperative Posturing Advice and Head Movements on the Progression of Macula-On Retinal Detachment
Source: Transl Vis Sci Technol. 2019 Mar 26;8(2):4. doi: 10.1167/tvst.8.2.4 (PMC6438104; doi:10.1167/tvst.8.2.4)
Supplement: Supplement 2 [file tvst-08-02-02_s02.pdf]

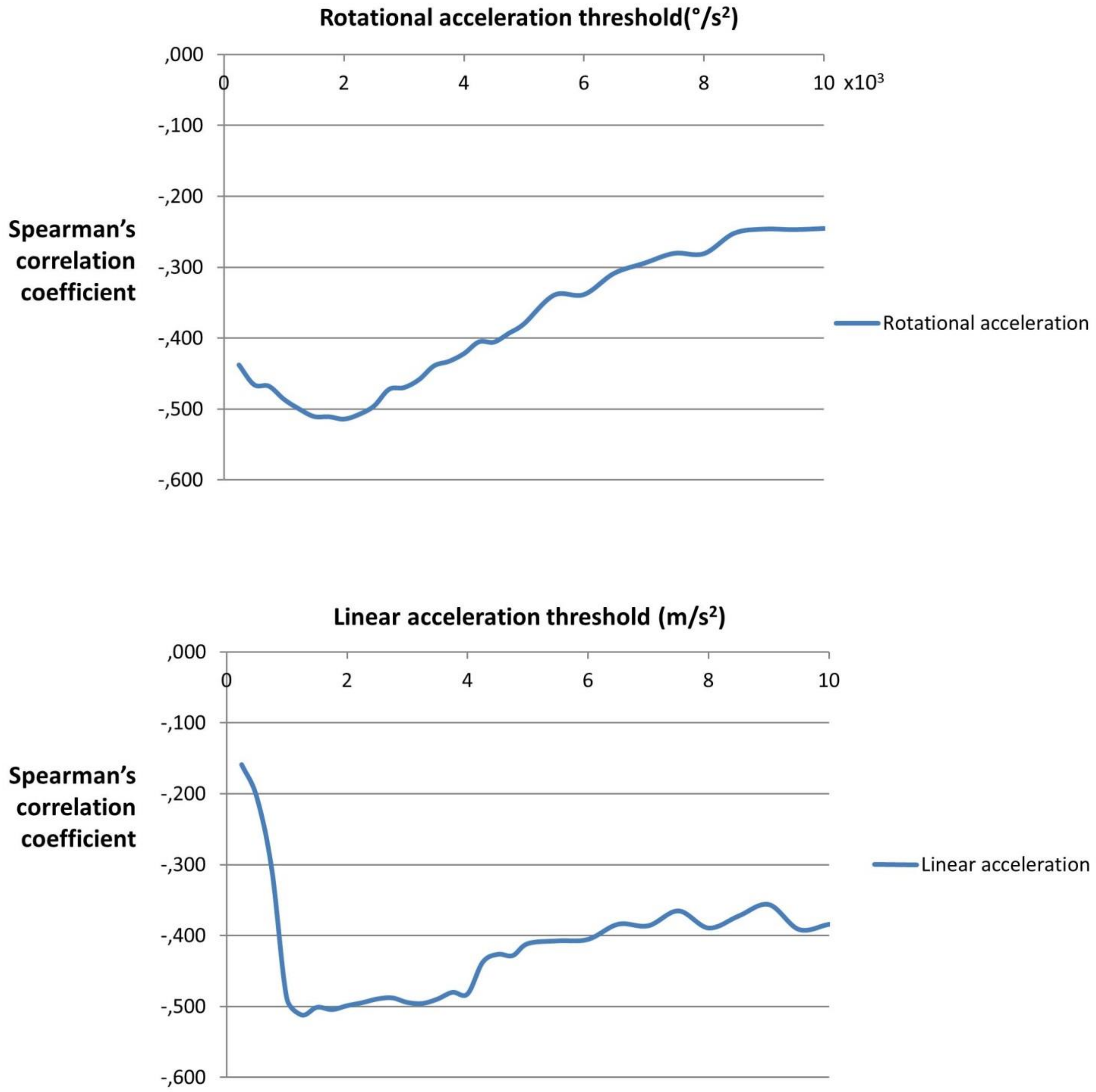

Supplemental figure 2. The correlation between RD progression and the average number of accelerations above various thresholds. The strongest Spearman's correlation coefficient found was -0.51 at a threshold-level of 2000 °/s^2 for rotational acceleration (top figure) and -0.51 at a threshold-level of 1.25 m/s^2 for linear acceleration (lower figure).
